# At-Risk Drinking Among Diabetic Patients

**DOI:** 10.4137/sart.s2243

**Published:** 2009-01-20

**Authors:** Susan E. Ramsey, Patricia A. Engler

**Affiliations:** 1 Department of Psychiatry and Human Behavior, The Warren Alpert Medical School of Brown University and Rhode Island Hospital, Providence, Rhode Island, U.S.; 2 Department of Medicine, The Warren Alpert Medical School of Brown University and Rhode Island Hospital, Providence, Rhode Island, U.S.

**Keywords:** at-risk drinking, diabetes, brief intervention

## Abstract

Diabetes Mellitus is a serious chronic disease, affecting an increasing number of individuals worldwide. Adherence to diabetes self-care behaviors is key to the successful management of the disease. At-risk drinking is common among diabetic patients and is associated with inferior diabetes treatment adherence and outcomes, resulting in increased mortality and morbidity. Furthermore, individuals with diabetes who engage in at-risk drinking are also in danger of incurring the negative consequences of at-risk drinking found in the general population. Research suggests that alcohol use screening and intervention do not commonly occur during the course of primary care treatment for diabetes. While methods for reducing alcohol use in this population have been largely unexplored to date, brief interventions to reduce at-risk drinking have been well-validated in other patient populations and offer the promise to reduce at-risk drinking among diabetic patients, resulting in improved diabetes treatment adherence and outcomes.

## Overview

The goal of this review is to provide the reader with an overview of the extant knowledge of the relationship between Diabetes Mellitus and alcohol use, with particular emphasis on the impact of at-risk drinking on diabetes. For the purposes of this review, searches were conducted on PsychInfo and PubMed.

## Prevalence and Consequences of Diabetes

Diabetes Mellitus (type 1 and type 2) is a serious chronic disease affecting 150 million people worldwide. 1 This figure is projected to double by the year 2025.^2^ In the United States, 7.8% of adults have been diagnosed with diabetes;^3^ however, this number is expected to increase rapidly due to the obesity epidemic.^1^ One report predicts that the prevalence rate of diabetes in the U.S. will more than double between the years of 2005 and 2050.^4^

If not well controlled, diabetes can result in significant morbidity and mortality; it is considered the sixth leading cause of death in the U.S.^3^ Multiple medical complications have been associated with diabetes including retinopathy, nephropathy, cerebrovascular complications, cardiovascular complications, peripheral vascular complications, and ketoacidosis.^1^ Indeed, diabetes is one of the primary causes of blindness, kidney failure, and lower extremity amputation.^5^ The aforementioned complications, along with certain psychosocial factors, are thought to exacerbate disability among people with diabetes;^1^ even “mild” diabetic complications have been shown to affect one’s quality of life.^6^

## Diabetes and Alcohol Use

Problematic alcohol use and diabetes commonly co-occur. The incidence rates for medical conditions such as diabetes are thought to be more than double among those with alcohol or drug problems relative to matched controls.^7^ Within one primary care sample, 28% of randomly selected adults with diabetes met criteria for a lifetime incidence of alcohol abuse, and 13% of the individuals with diabetes met either current (1%) or lifetime (12%) criteria for alcohol dependence.^8^ The rates were higher among those with co-morbid diabetes and hypertension; 38% met for either current (3%) or lifetime (35%) alcohol abuse, and 16% met criteria for either current (4%) or lifetime (12%) alcohol dependence.^8^ Fleming and Mundt^8^ suggest that this translates into 1.35 million individuals with diabetes in the U.S. who “drink too much.”

Rates of problematic drinking are similar among other samples of individuals with diabetes. During the course of one study of veterans receiving medical treatment, 17.8% of patients treated for diabetes also received treatment for alcohol dependence. 9 High rates of co-morbid diabetes and alcohol problems have also been found among adolescents. For instance, in a study of adolescents at a camp for individuals with diabetes, Gold and Gladstein^10^ found that 24% of the campers “drank dangerously.” Significant substance use among individuals with diabetes has been found in other medical samples. For example, in one study of individuals with diabetes seeking treatment for severe hypoglycemia, 17% had been drinking, and 31% had been using some type of drug or alcohol.^11^

## Impact of Alcohol Use on Diabetes

The relationship between alcohol use and diabetes is quite complicated. In regard to the onset of diabetes, light to moderate alcohol use has been found to be associated with a lower incidence of diabetes while heavy drinking (commonly defined as ≥48 g/day or ≥6 drinks per day) has been linked to an increased risk for the onset of diabetes.^3^,^12^,^13^ There is also some evidence that light to moderate drinking (defined as ≤2 drinks per day) can have beneficial effects on glycemic control in nondiabetic populations.^14^,^15^

In individuals with diabetes, the short-term impact of alcohol use is an area of research that has produced very mixed results. Differences among studies, such as whether alcohol is administered with or without a meal and whether a fasting glucose level is measured, make comparisons across studies difficult.^3^ Alcohol consumption is believed to impact glycemic control and may impair glucose production when used excessively.^16^,^17^ Some research has found that alcohol consumption may induce hypoglycemia.^18^,^19^ It has been argued that using even small amounts of alcohol can have deleterious effects on glucose control;^20^ however, a recent study found no acute effect of small doses of alcohol on plasma glucose or serum insulin.^21^ Finally, alcohol may interact with hypoglycemia to produce significant increases in diastolic blood pressure or exacerbate cognitive deficits associated with hypoglycemia.^22^

The longer-term impact of alcohol use on diabetes course and sequelae has also been examined. In a recent study, Ahmed and colleagues^23^ found that higher levels of alcohol use were associated with lower hemoglobin A_1c_ (HbA_1c_) levels through a nadir of 2–2.9 drinks per day. HbA_1c_, a widely used measure of glycemic control, is an important predictor of morbidity; it has been associated with greater medical complications and greater disability.^24^–^28^ In another recent study,^29^ individuals with diabetes who had abstained from alcohol use were randomly assigned to consume one glass of wine or one nonalcoholic beer per day. After three months, those who had consumed one alcoholic drink per day had a lower fasting glucose level. However, the groups did not differ on postprandial glucose levels. In a similar study, individuals with diabetes who drank 1–2 glasses of wine per day for 30 days had lower fasting serum insulin but had no change in fasting plasma cholesterol, HDL cholesterol, glucose, or HbA_1c_ relative to a 30-day period of abstinence from alcohol.^21^ A meta-analysis of the relationship between alcohol use and coronary heart disease and mortality in individuals with type 2 diabetes found that rates of coronary heart disease and coronary heart mortality are significantly lower in all three categories of alcohol consumers examined, compared to non-drinkers.^30^ In addition, the risk of total mortality was significantly lower in the lightest drinking group, compared to non-drinkers. It is of note that the lower limit of the highest drinking category examined in this study was only 1.5 drinks per day. Drinking rates that exceed that level have been found to be associated with greater risk of total mortality, as well as greater risk of coronary heart mortality, for individuals with diabetes.^30^,^31^ Furthermore, heavy alcohol use has been associated with diabetic neuropathy and retinopathy.^19^ Alcohol consumption is also thought to interfere with neuroendocrine, gastrointestinal, and sexual functioning.^20^ One study found that there were greater rates of atherosclerosis between heavy and non-drinkers compared to light drinkers. 32 Another found that heavy alcohol consumption interacted with diabetes to increase risk for hepatocellular carcinoma.^33^

In addition, there is some evidence to suggest hazardous interaction effects between alcohol and diabetes medications. Although one study reported no serious adverse events in response to alcohol ingestion while taking troglitazone,^34^ negative interaction effects between alcohol and other diabetes medications have been found. For instance, the likelihood that alcohol will induce hypoglycemia is greater in the presence of sulphonylurea medication.^19^ Another medication, the sulfonylurea derivative chlorpropamide, was found to decrease the rate of ethanol elimination from the blood.^35^ Additionally, experts caution against using alcohol excessively for patients taking metformin.^36^

## Adherence to Diabetes Treatment Recommendations and the Effect of Alcohol Use on Adherence

Effective diabetes treatment requires considerable patient involvement.^5^ Self-care behaviors have been described as the cornerstone of diabetes treatment, significantly impacting disease course.^37^ Glucose self-monitoring,^38^,^39^ appropriate diet,^40^ and exercise,^41^,^42^ along with compliance with prescribed medications^43^,^44^ and attendance at medical appointments, 45,46 have all been associated with better glycemic control, as measured by HbA_1c_.

Despite the importance of adhering to treatment recommendations, many individuals with diabetes fail to do so. For instance, in one study of individuals with type 2 diabetes in Mexico, only 26% of patients followed three main treatment recommendations including medication compliance, meal planning, and exercise.^47^ Other research has identified barriers to glycemic control among college students with type 1 diabetes;^48^ barriers included alcohol use. Similar barriers emerge cross-culturally. In one study of Finnish adolescents with insulin-dependent diabetes, Kyngas^49^ found that only 19% of diabetic participants reported “good” adherence to treatment recommendations. Alcohol use was significantly associated with poor adherence.

Research has shown that individuals with diabetes who drink alcohol are at increased risk for poor adherence regarding many important health behaviors. For instance, alcohol use may decrease one’s food intake^16^ or reduce patients’ willingness to adhere to dietary regimens.^20^ Further, researchers argue that because alcohol impairs judgment, it interferes with one’s attention to diet and medication^20^ and may adversely affect other self-care behaviors such as exercise and glucose self-monitoring.^3^,^50^ Heavy drinkers have been shown to have poorer insulin treatment adherence and reduced motivation to adhere to their treatment regimen.^20^ Poor treatment adherence has also been demonstrated among those who drink moderately. Chew and colleagues^51^ found that both heavy and moderate drinkers with diabetes were less likely to perform daily glucose self-monitoring and less likely to have medical provider visits, when compared to non-drinkers. That is, heavy drinkers were less likely to have eye examinations, and moderate drinkers were less likely to have had any provider visit in the previous year.

In the largest study of self-care behavior of diabetic primary care patients, Ahmed and colleagues examined the impact of alcohol use on self-care.^37^ This diverse and representative sample of nearly 66,000 patients was drawn from multiple hospitals and outpatient clinics. Data were collected regarding average number of alcoholic beverages consumed per day, as well as adherence to six important diabetes self-care behaviors including exercising, smoking, blood glucose self-monitoring, taking diabetes medications, following a healthy diet, and annual HbA_1c_ testing. The results indicated that more than half of diabetic patients reported current alcohol use. Heavy drinkers had the highest rates of additional morbidity such as peripheral neuropathy. Importantly, Ahmed and colleagues found a significant negative association between alcohol use and diabetic self-care behavior, indicating that greater alcohol use was significantly associated with poorer diabetes treatment adherence. This negative association between alcohol use and self-care behavior started at the level of one drink per day. This association between poor self-care behavior and increased alcohol consumption was found across all six diabetes self-care behaviors examined in this study.

Similar findings have been demonstrated among primarily ethnic minority samples. Johnson and colleagues^52^ found that diabetes self-care behaviors were affected by alcohol use within a sample of mostly Hispanic (61%) and African American (29%) primary care patients. That is, any recent drinking was significantly associated with poorer adherence to dietary, exercise, and medication recommendations as well as with poorer attendance at follow-up appointments.

## Other Consequences of At-Risk Drinking

In addition to the impact of alcohol use on diabetes management and morbidity, at-risk drinking can result in other negative consequences for individuals with diabetes. Within the general population, a significant proportion of alcohol-related problems, such as injuries, health problems, and psychosocial problems, occur among non-dependent drinkers.^53^ For example, deaths from any type of cirrhosis are more likely to be found among individuals who drank three or more drinks per day, on average,^54^ and averaging more than 5 drinks per day is associated with significantly increased rates of diseases of the digestive, respiratory, and circulatory systems and malignant neoplasms, relative to individuals drinking no more than 2 drinks per day.^55^ Furthermore, the likelihood of death before the age of 65 increases with each drinking category, from light drinking (up to 3 drinks per week), to moderate drinking (between 3 and 14 drinks per week), to heavy drinking (14 or more drinks per week).^56^ Kranzler and colleagues^57^ found increased risk of a variety of medical and psychosocial problems among men who averaged 40–80 grams of ethanol per day (approximately 3–6 drinks) and women who averaged 25–65 grams of ethanol per day (approximately 2–5 drinks). Mertens and colleagues^58^ describe similar findings, in their large primary care sample of at-risk “hazardous” drinkers identified through elevated hazardous drinking screening test scores. Hazardous drinkers incurred greater total medical costs than non-hazardous drinkers. Also, they were significantly more likely than non-hazardous drinkers to experience certain physical problems (e.g. chronic obstructive pulmonary disease), psychological problems (i.e. anxiety disorders), injuries and overdoses.^58^ Those who drink at low or moderate volumes annually are responsible for the majority of alcohol related negative consequences including fights and hospitalizations for suicide attempts or violent injuries.^59^ Similar findings have been reported elsewhere.^60^

Individuals who report heavy drinking days are particularly at risk for incurring negative consequences. Midanik and colleagues^61^ found that those who reported fewer than 5 heavy drinking days (5 or more drinks in a given day) in the past year comprised 35% of the individuals in the sample who reported driving while intoxicated; individuals who reported 25 or fewer heavy drinking days in the past year comprised 68% of the individuals who reported driving while intoxicated. Furthermore, individuals who reported one or more heavy drinking days in the past year had a significantly greater risk of having a symptom of alcohol dependence, having an alcohol-related work problem, or having driven while intoxicated, compared to individuals with the same average alcohol intake who reported no heavy drinking days in the past year.^61^ More recently, Dawson and colleagues^62^ reported a similar increased risk of alcohol abuse and/or dependence with an increased frequency of heavy drinking days. Other investigators^63^ have reported a similar steep rise in alcohol-related problems with increasing frequency of heavy drinking days. In one large, nationally representative sample of college students, Presley and Pimental^64^ found a sharp increase in negative consequences based on the frequency of heavy drinking episodes. Nonheavy drinkers averaged four negative consequences in the last year whereas heavy drinkers experienced 12. Heavy and frequent drinkers experienced 28 negative consequences in the last year. Further, the heavy and frequent drinkers were shown to have nearly twice as much alcohol in their bloodstream during an average drinking episode compared to heavy drinkers (7.5 residual drinks versus 4.3).^64^ Given the impact of at-risk drinking in the general population, there is a need for more research on this topic among individuals with diabetes.

## Recognition and Treatment of Alcohol Problems in Primary Care

Primary care appointments provide an opportunity to assess for problematic alcohol use. This is especially true for alcohol use that may impact medical treatment of conditions such as diabetes.^65^ However, research indicates that physicians are poorly informed about how alcohol use affects the management of diabetes.^3^ Additionally, there appear to be several barriers to adequate assessment of and intervention regarding alcohol use including problem recognition and knowledge of appropriate intervention. One report suggested that few primary care patients are screened for alcohol use and that those who are tend to possess certain characteristics such as a psychiatric diagnosis.^66^ It is important to note that screening for at-risk drinking can be done very simply by asking patients how often they consume alcohol and how many drinks they typically have when they do consume alcohol. Further, fewer than half of identified problem drinkers received any follow-up on this issue. The most common intervention appeared to be being told to “stop drinking” from the primary care provider.^66^ Another study queried general practitioners and psychiatrists regarding alcohol and drug use among their patients. This study revealed considerable misconceptions about empirically supported substance use treatments, suggesting that practitioners were misinformed about which treatments are effective.^67^ Physicians reported that alcohol screening was more problematic than nicotine screening because of multiple barriers including problem recognition, perceived importance of alcohol use, inadequate intervention tools, feared stigmatization, and physicians’ expectations about the effectiveness of intervening.^68^ More specifically, only 6% of the physicians studied were aware of established (Finnish) guidelines concerning heavy drinking. They tended to believe that patients would conceal their alcohol use and, as a result, were more likely to discuss alcohol use only under certain circumstances such as when a patient “smelled of alcohol.” In addition, physicians tended to minimize the potential risks of alcohol use, with some citing its possible medical benefits.

The physicians in this study^68^ indicated that limited or ineffective treatment tools diminished their willingness to intervene with patients’ alcohol use. Further, they suggested that they would be more likely to address alcohol abuse issues if there were more effective medications, “patches,” or pamphlets with quit strategies that could be utilized with interested patients. Despite some willingness to intervene with interested patients, many physicians seemed quite hesitant to raise the issue of alcohol themselves. In some cases, the physicians reported reluctance to record even obvious alcohol problems out of concern that this might offend the patients. Furthermore, they seemed to think that advising patients on alcohol use was too time consuming and ineffective. However, the authors suggest that this may be due to a lack of follow-up appointments regarding recommended alcohol consumption reductions that could potentially provide the physicians with feedback about more promising results.

In a similar qualitative study, Beich and colleagues^69^ obtained feedback from general practitioners regarding their experiences with a screening and brief intervention for excessive alcohol use. The physicians in this study were surprised by how difficult they found it to be to discuss positive screening results with their patients. Some of the physicians reported that they did not feel confident in their ability to provide effective counseling regarding lifestyle issues to their patients. It was also common for the physicians to express reluctance to discuss excessive alcohol use with their young patients, whose alcohol use was seen as a phase which they would outgrow.

## Brief Interventions to Address At-Risk Drinking

The empirical literature provides a strong basis for the potential of brief interventions to reduce at-risk drinking. In McCrady’s^70^ review of treatments for alcohol abuse and dependence, brief intervention was one of only two treatments that met criteria for “efficacious” treatment. Similar conclusions have been reached in other reviews,^71^–^75^ and numerous studies have provided empirical support for the efficacy of brief interventions.^76^–^83^

Furthermore, brief alcohol interventions show considerable promise in primary care settings, with several systematic reviews demonstrating their efficacy in this setting. For example, Richmond and Anderson^84^ examined alcohol interventions for at-risk drinkers provided by general practitioners. Brief alcohol interventions led to significantly better treatment outcomes than standard care; “very brief advice” resulted in reductions in both drinking volume (25%–35%) and the proportion of “excessive drinkers” (45%). Goldstein and colleagues^65^ reviewed health behavior interventions conducted in primary care settings and concluded that brief interventions to reduce risky or harmful drinking demonstrated efficacy. Two methodologically rigorous meta-analyses also support this finding. Moyer and colleagues^74^ found small to medium effect sizes for brief alcohol interventions relative to control conditions among primary care patients not seeking treatment for alcohol issues. In a recent meta-analysis, Bertholet et al.^77^ examined the efficacy of brief alcohol interventions to reduce longer term alcohol use among primary care patients. Their results indicate that brief interventions significantly reduced alcohol consumption relative to controls and that the treatment effects held through a 12-month follow-up period.

Based on a systematic review of behavioral counseling interventions, the United States Preventive Services Task Force recommends screening and brief interventions for problematic drinking in primary care settings.^83^,^85^ They concluded that brief interventions led to statistically significant reductions in alcohol use if the interventions incorporated two or more of the following elements: feedback regarding drinking, advice to reduce drinking, and goal setting.

One element of brief intervention that shows considerable promise in the primary care setting is brief advice. A preponderence of studies have demonstrated that brief advice delivered by treatment providers in the context of a medical visit is effective in reducing hazardous alcohol use. In an early study, Anderson and Scott^76^ found that men classified as heavy drinkers in primary care clinics benefited from receiving brief advice from their own general practitioner relative to controls. Intention to treat analyses revealed that treatment group participants reported significantly greater reductions in their alcohol use relative to controls at a 12-month follow-up. Two 10- to 15-minute physician-delivered interventions involving education and advice to reduce drinking have also proven effective.^79^,^80^ Relative to controls, this intervention led to significant reductions in mean number of drinks and frequency of excessive drinking during the previous 7 days, as well as a reduction in binge drinking episodes during the previous 30 days. These results were maintained from the 6-month to the 12-month follow-up^80^ and held through a 48-month follow-up.^79^ In addition, this intervention resulted in a favorable benefit-cost ratio. The benefit-cost ratio for the intervention was 4.3 to 1 for medical care savings (e.g. fewer emergency room visits and hospitalizations) and 39 to 1 for “societal” savings (e.g. fewer legal events and motor vehicle accidents). Further benefit-cost research from this group has also yielded promising results. Fleming and colleagues^86^ found that the economic benefit of their primary care brief intervention was $1151 per patient. They cite the benefit cost ratio as 5.6 to 1. Similar findings have been cited by other groups.^87^

Positive results have been obtained with even briefer alcohol interventions. One study found support for a 5–10 minute “counseling session” containing advice for drinking goals delivered by primary care providers as part of a routine medical visit. Among high-risk drinkers, this intervention led to significant reductions in alcohol use at a 6-month follow-up relative to controls.^81^ Such brief interventions may be more time- and cost-effective than more lengthy treatments. For instance, a comparison of general practitioner delivered brief advice with a three-session intervention and a seven-session intervention yielded no significant differences in drinking outcomes.^88^ During the three-year follow-up period, there were no significant differences among groups in weekly drinking amount, drinking occasions per week, and usual drinking amount per occasion. There was not a no-treatment control group in this study.

## Brief Alcohol Interventions with Diabetic Patients

To date, only one published study has tested a brief intervention for alcohol use among diabetic patients. Fleming and colleagues^8^ evaluated the efficacy of a brief intervention to reduce alcohol use among type 2 diabetic or hypertensive patients in a primary care setting.^78^ This intervention consisted of brief advice in the form of two 15-minute sessions conducted by either a nurse practitioner or physician assistant as well as two 5-minute follow-up telephone calls from an office nurse. The intervention included feedback containing test results of an alcohol biomarker, carbohydrate-deficient transferrin (CDT).

The study results indicated that significantly more intervention participants reduced heavy drinking and CDT levels from baseline to followup compared to control participants. This study shows considerable promise for the efficacy of brief alcohol interventions among diabetic patients in primary care. However, there are certain factors that limit the conclusions that can be drawn from the results of this study. First, diabetic outcome variables were not explored. The impact of alcohol use on diabetic treatment adherence and outcome is an area in need of further examination. Second, participants with significant levels of CDT who did not report hazardous drinking were included. The authors acknowledged the possibility of these “false positives” as a study limitation. Third, the selected drinking cutoffs for study inclusion did not correspond with “at-risk” drinking cutoffs established by the National Institute on Alcohol Abuse and Alcoholism.^89^ Fourth, diabetic and hypertensive patients were both included in the sample, making it difficult to discern the impact of the intervention among only individuals with diabetes. Fifth, the intervention did not include the provision of information regarding the specific effects of at-risk drinking on diabetes.

## Conclusions

Diabetes and at-risk drinking are major public health issues. At-risk drinkers represent a significant subpopulation of diabetic patients who, in the absence of a change in drinking, are likely to have poor diabetes treatment adherence and outcomes, resulting in increased morbidity and mortality. Furthermore, they are at risk for additional adverse drinking-related consequences. We believe that current research and practice have devoted insufficient attention to screening for and addressing at-risk drinking among diabetic patients. Brief interventions to reduce at-risk drinking have been well validated in a variety of patient populations and offer the promise of improving diabetes treatment adherence and outcomes.
